# Nutrient gaps and dietary adequacy among adolescent girls in rural North-Eastern Ghana: the role of local food-based approaches, school lunch and multiple-micronutrient fortified biscuits

**DOI:** 10.1017/S0007114525103929

**Published:** 2025-07-28

**Authors:** Fusta Azupogo, Karin J. Borgonjen-van den Berg, Richmond Aryeetey, Inge D. Brouwer

**Affiliations:** 1 Institute for Global Nutrition and Department of Nutrition, University of California, Davis, USA; 2 Department of Family and Consumer Sciences, Faculty of Agriculture, Food and Consumer Sciences, University for Development Studies, Box TL 1882, Tamale, Ghana; 3 Division of Human Nutrition and Health, Wageningen University and Research, Wageningen, The Netherlands; 4 School of Public Health, University of Ghana, Legon, Accra, Ghana; 5 International Food Policy Research Institute, Washington, DC, USA

**Keywords:** Adolescent girls, Nutrient gaps, Dietary adequacy, Multiple-micronutrient fortified food, School lunch, Food-based recommendations, Optifood, Ghana

## Abstract

A local food-based approach, including school lunch with multiple-micronutrient fortified biscuits (MMB) as supplementary snacks, may enhance dietary adequacy, although current evidence remains limited. This study assessed nutrient inadequacies and developed food-based dietary recommendations (FBR) incorporating school lunch from the Ghana School Feeding Programme (GSFP) and MMB. Data from 292 girls aged 10–17 years, enrolled in the Ten2Twenty-Ghana study was analysed. Dietary intake was assessed via a quantitative 24-h dietary recall. Usual intakes were estimated using the National Cancer Institute method. Linear programming with Optifood was used to develop FBRs based on commonly consumed foods (≥5% of participants) and their median serving sizes, intake frequency, nutrient content, and cost per 100 g. Constraints included estimated energy needs and harmonised average nutrient requirements. The mean usual energy intake was 2351 (sd 66) kcal/d. Ca (99·8 %), vitamin B_12_ (99·8 %), riboflavin (96·2 %), vitamin A (91·5 %), vitamin C (87·6 %), Fe (73·7 %), folate (49·3 %) and Zn (8·5 %) inadequacies were prevalent. Optimised diets achieved adequacy for protein and most micronutrients, except Ca and vitamin B_12_, besides vitamin A for 15–17-year-old girls. School lunch from the GSFP did not enhance micronutrient levels when added to the daily diet. Adding MMB to the daily diet ensured adequacy for vitamin C, riboflavin and Fe, although marginal for Fe. Ca and vitamin A improved substantially with MMB for girls aged 15–17 but remained below the harmonised average requirements. Integrating regular school lunch with specialised fortified foods may be a cost-effective strategy to enhance dietary adequacy for adolescent girls in rural areas.

The number of adolescents globally is projected to be higher in sub-Saharan Africa than in any other region by 2050^(1)^. Yet, sub-Saharan Africa has the worst adolescent health profiles, with persistently high mortality from maternal and infectious causes^(2)^. While prevention of stunting in the first 1000 d remains a priority, adolescence provides a second (and last) window of opportunity for high returns on investment with nutritional interventions, including those implementing food-based dietary recommendations (FBR)^(1,3,4)^.

Inadequate dietary intake evolving from food insecurity and the intake of monotonous plant-based diets with little or no animal-sourced foods (ASF) partly explains why adolescents in low- and middle-income countries have insufficient micronutrient intake and, consequently, high rates of anaemia and Fe deficiency^(5,6)^. Plant-based diets are high in concentrations of phytates and other dietary inhibitors, leading to reduced bioavailability of micronutrients; this results in nutrient inadequacies^(7,8)^ with consequences for anaemia and Fe deficiency anaemia^(5,6)^. In Ghana, undernutrition and micronutrient deficiencies are prevalent among adolescent girls^(9–12)^. Particularly in the northern regions, anaemia and other micronutrient deficiencies are notably widespread; here, the prevalence of anaemia is a significant public health concern^(13,14)^.

Although little is known about nutrient gaps in adolescent girls’ diets in low- and middle-income countries, dietary inadequacies of multiple micronutrients, such as Fe, Ca, Zn, folate and vitamins A and C, were reported for Senegalese adolescents, particularly for deficits in folate and Zn intake^(15)^. In Ghana, Annan *et al.* ^(16)^ estimated that about half of girls aged 9–13 years in the Kumasi Metropolis of Ghana have inadequate intakes of vitamin A and folate, with at least one-third having dietary inadequacies of vitamin B_12_, Zn and Fe. Further, a study among 5–13-year-old school-aged children in northern Ghana confirms the existence of dietary inadequacies of multiple micronutrients^(17)^.

For girls, nutritional problems may be perpetuated by gender bias in diet, education and aspirations^(18)^. For instance, girls in Ghana are disadvantaged in intra-household food distribution and resource allocation^(19)^. The pubertal stage of adolescent girls may influence their dietary habits and patterns. For instance, mid-adolescent girls in India were found to consume less protein and fewer vitamin-rich foods compared with early adolescent girls^(20)^. To the best of our knowledge, no study has examined the differences in nutrient gaps or formulated FBR with considerations for the pubertal stage of adolescents. However, Oy *et al.* ^(21)^ formulated FBR tailored to anaemic and non-anaemic adolescent girls in Indonesia.

Consuming multiple-micronutrient-fortified foods improves adolescents’ haematologic and micronutrient status^(22,23)^. Consequently, including multiple-micronutrient fortified snacks such as biscuits (MMB) in FBR may be crucial in ensuring dietary adequacy for school children and adolescents in low socio-economic contexts with high food insecurity and dietary inadequacies. Yet, there is limited evidence on this approach. School feeding is an essential global intervention programme designed to improve attention in school, learning and the nutrition and health of children in low socio-economic contexts^(24)^. The Ghana School Feeding Programme (GSFP), initiated in 2005, has since been expanded to cover over 9000 underprivileged rural primary schools nationwide^(25)^. The GSFP has been shown to improve nutrient intake and height-for-age z-score (HAZ) of school-aged children, particularly girls in low socio-economic contexts^(17,26)^.

The recent introduction of food-based dietary guidelines (FBDG) in Ghana broadly targets the adolescent and adult populations but lacks specific considerations for rural adolescent girls^(27)^. The primary aim of the present study was to assess nutrient inadequacies and develop and evaluate alternative local FBR tailored to meet the nutrient requirements of adolescent girls in North-Eastern Ghana. We also evaluated whether including a fortified food product such as MMB in local FBR with (out) observed school lunch (SL) from the GSFP improves dietary adequacy. The FBR from the present study may help ensure dietary adequacy for adolescent girls in rural contexts such as northern Ghana. Our findings may also help inform decision-making in improving the GSFP and future revisions of the Ghana FBDG for populations at risk of dietary inadequacies in low socio-economic contexts.

## Methods

### Study design and participants

Details of the study design, population, sample selection and methods have been described elsewhere^(28)^. In brief, the present study was based on cross-sectional dietary intake data collected before a 26-week randomised placebo-controlled trial (*n* 621) of the efficacy of MMB on the micronutrient status of adolescent girls aged 10–17 years in the Mion District of Ghana^(28)^. In that trial (Ten2Twenty-Ghana), the girls consumed either a pack (51·3 (sd 3·2) g) of MMB or unfortified biscuits 5 d weekly as a snack between March and August 2019. The MMB was fortified with eleven vitamins, including thiamine, riboflavin, B_6_, B_12_, A, D, K_1_, E, niacin, folic acid and ascorbic acid, and seven minerals, including Zn, Ca, Fe, Cu, iodine, Se and Mg (online Supplementary Table S1). The fortification levels of the MMB were designed to provide 15 and 30 % of the RDA for fortified minerals and vitamins, respectively, for young women aged 19–30 years.

The study population in the trial included apparently healthy, non-pregnant and non-breastfeeding pre- and post-menarche girls selected from nineteen primary schools across the district. Ten (10) out of the nineteen schools participated in the GSFP at the time of the survey. A subset of the girls (*n* 299) enrolled in the trial was randomly selected for a quantitative 24-h dietary recall (24HR) to quantify food intake in and outside the home before the run-in to the trial. We further selected a random sample of 100 (∼33 %) girls out of the 299 first 24HR for a repeated 24HR to adjust for the random day-to-day variation in dietary intake. The repeated 24HR was conducted on non-consecutive days to avoid dependency of dietary intake on the 2 d.

The sample size for the 24HR was calculated using the one-random sample formula, considering a 95 % CI. For Fe intake, an estimated width of 10·1 mg and an sd of 28·9 mg^(29)^ were used. For vitamin A intake, an estimated width of 50·5 µg retinol equivalents and an sd of 113·2 µg retinol equivalents^(29)^ were used. The estimated required sample was 130 girls, rounded up to 150, accounting for a 20 % non-response rate. Following Rothman’s^(30)^ recommendation, a minimum subsample of fifty girls was required for the repeated recalls. The sample size is comparable to previous studies using the linear programming approach in sub-Saharan Africa^(31,32)^. This study was conducted in accordance with the guidelines of the Declaration of Helsinki, and all procedures involving human subjects were approved by the Navrongo Health Research Centre Institutional Review Board (NHRCIRB323). Written approval was granted by the Ghana Education Service, and informed consent was obtained from the leaders of participating communities. Participation was entirely voluntary, with each girl providing assent after her parent or guardian had given signed or thumb-printed informed consent.

### Data collection

The data were collected over 2 weeks from Friday, 14 December to Thursday, 20 December 2018, and from Friday, 18 January to Thursday, 24 January 2019. A decision was taken to suspend the work during the Christmas and New Year festivities and until at least a week after schools resumed, ensuring that we did not capture festive foods; this also enabled us to capture any foods consumed through the GSFP. Trained enumerators with a first degree in nutrition who spoke the local languages (Dagbani or Likpakpa) collected the data. Trained supervisors with prior experience in dietary assessment observed a random selection of the interviews and validated the survey forms in the field. In the case of incompleteness or inconsistencies, households were revisited, and corrections were made. Before data processing and analysis, all data entries were verified.

### Quantitative 24-h dietary recall

We assessed the current intake of the girls with a quantitative 24HR using the US Department of Agriculture standard multiple-pass procedure^(33)^. The details of the 24HR have been described elsewhere^(34)^. In brief, subjects were asked to mention all foods (including drinks and snacks) consumed in the last 24 h (wake-up to wake-up). They were then asked to describe the ingredients and cooking methods for mixed dishes in detail. We recorded the weight of each food item, beverage and ingredients of mixed dishes with duplicate portions of foods consumed with a digital kitchen weighing scale (Soehnle Plateau, model 65086), precisely to 2 g, with a maximum capacity of 10 kg. In the absence of duplicate portions, we estimated in priority order: the monetary value, the weight equivalent with other food items (e.g. sugar with corn flour), volume, food models (e.g. small, medium, large) or household measures (e.g. spoon, ladle). The weight of ingredients in mixed dishes was estimated by multiplying the proportion of the recipe consumed by the weight of the ingredients used in cooking the recipe. The proportion consumed was estimated by calculating the ratio of the volume consumed by the girl to the total volume of food cooked. For shared bowl eating, the girl’s usual intake was estimated by asking her to report the quantity she typically consumes of the specific food or dish when eating alone. Finally, we recorded the frequency of intake for each food item in the last week. The interview ended with probing for likely forgotten foods, mainly fruits, sweets, beverages and snacks consumed on the recall/previous day.

### Market survey and development of conversion factors

We developed standard recipe data for food recipes that were bought and consumed, as well as foods eaten through the GSFP, following the guidelines of Gibson and Ferguson^(33)^. The standard recipe data was used to estimate the weight of ingredients consumed from bought recipes and school-feeding recipes. Additionally, a market survey was conducted in four different markets from four different localities in the district. The market survey data were used to estimate the mean price in Ghana Cedis (GH¢) per 100 g of edible food for each listed food item in the recalls and to develop conversion factors for the monetary value-to-weight of each food item and bought standard recipes. Finally, weight-to-weight, volume-to-weight, food model-to-weight, household-measure-to-weight conversion factors and waste factors were developed following the recommended procedures^(33)^.

### Nutritional status and household characteristics

Before the 24HR, the adolescent girls’ height (cm) and weight (kg) were measured according to the standard guidelines^(35)^. HAZ and BMI-for-age z-score (BAZ) were calculated with WHO AnthroPlus using the WHO growth reference for 10–19-year-old girls. Stunting was defined as HAZ < –2 sd, whereas BAZ was categorised as thinness (BAZ < –2 sd), normal weight (–2sd ≤ BAZ ≤ +1 sd) and overweight/obese (BAZ > +1 sd)^(36)^. Professional phlebotomists from the Tamale Teaching Hospital assessed Hb by finger prick using a HemoCue 301 (Angelholm, Sweden; 0·1 g/dl precision). The photometer was calibrated using certified quality control samples from the CDC/Atlanta, and ten girls’ measurements were repeated daily for quality control. Anaemia was defined as Hb < 120 g/l for girls aged ≥ 12 years and < 115 g/l for girls aged < 12 years^(37)^.

An index of household wealth (range: 25–100) was computed based on the international wealth index^(38)^. The index uses household ownership of durable assets, access to electricity, the type of water and toilet facilities accessed by the household and the floor material of the household. The severity of household food insecurity, categorised as food secure, mild, moderate or severe food insecurity, was assessed using the Food Insecurity Experience Scale^(39)^. Based on data from a household roster, household dependency ratios, sex and literacy ratios were computed similarly to the Ghana Statistical Service^(40)^.

### Energy and nutrient intake estimation

The nutrient calculation system Compl-eat^TM^ (version 1.0) of Wageningen University and Research was used to estimate energy and nutrient intakes, including carbohydrates, fat, protein, thiamine, riboflavin, niacin, vitamin B_6_, folate, vitamin B_12_, vitamin A, vitamin C, Fe, Zn and Ca. We updated and used a food composition table specifically created for a food intake survey in Ghana^(31,41)^. The general Atwater factors – 17 kJ/g (4·0 kcal/g) for protein and carbohydrates and 37 kJ/g (9·0 kcal/g) for fat^(42)^ – were considered for the calculation of the energy content of foods. The metabolisable energy factor for dietary fibre (8·0 kJ/g /2·0 kcal/g) recommended by the FAO^(43)^ was also used to calculate energy intake. Total vitamin A (retinol activity equivalent) intake was calculated as the sum of retinol and 1/12 *β*-carotene^(44)^. Retention factors of the US Department of Agriculture^(45)^ were used to calculate the nutrient values of cooked food.

### Usual intake and probability of inadequacy

The z-score approach was used to identify outliers (*n* 7) in energy intake, and participants were removed from the sample for analysis when their z-score for energy intake was improbable (z-score < –3 or z-score > 3)^(46)^. We adjusted energy and nutrient intake for random day-to-day (within-person) variation in intake using the Simulating Intake of Micronutrients for Policy Learning and Engagement SAS macros^(47,48)^. These macros, designed to estimate usual intake, simplify the US National Cancer Institute methodology^(49)^. The probability of nutrient inadequacy was estimated as the percentage of participants whose intake fell below the harmonised average requirements (H-AR) suggested by Allen *et al.* ^(50)^ for protein and eleven micronutrients, including Fe, Zn, Ca, vitamins A and C, thiamine, riboflavin, niacin, folate and vitamins B_6_ and B_12_. For Fe, we utilised the full probability approach^(51)^, considering a 5 % bioavailability due to the low dietary haem Fe and high levels of phytates and fibre in the commonly consumed plant-based foods. For Zn, the requirements were defined by the estimated average requirements (EAR) from the International Zinc Nutrition Consultative Group for unrefined cereal-based diets, which presumes a bioavailability of 30 %^(52)^. The usual intake analyses were adjusted for several covariates, including the girl’s age, household wealth and food insecurity, whether the intake was assessed on a weekday or weekend, the specific day of intake, whether the intake day was a festive day and any reported sickness on the intake day. Two hundred bootstrap resampling replicates were used to derive 95 % CI and standard errors. Habitual intakes were presented as the mean and standard error. The mean probability of adequacy for the eleven micronutrients was determined by averaging the individual probabilities of adequacy across all micronutrients. The probability of adequacy is the reverse of inadequate intake (inadequacy), defined as the percentage of the population with intakes ≥ the H-AR; together, the probabilities of adequacy and inadequacy sum up to 100 %. The usual intake and probability of (in)adequacy analysis was conducted in SAS 9.4 (SAS Institute Inc.).

### Food-based dietary recommendations development

Optifood linear programming tool (version 4.0.9)^(53,54)^ was used to develop and evaluate the FBR. Optifood is a four-module linear programming approach to formulate FBR^(54,55)^. The approach predicts if a local food-based approach or additional approaches such as SL or special fortified food products are required to ensure nutrient adequacy for high-risk populations and the extent to which these measures might contribute to its achievement^(56)^. The Optifood model parameters were based on the first 24HR data and were defined using Microsoft Excel 365, IBM SPSS (Version 25) and a pre-designed Microsoft Access 2010 template for Optifood^(57)^. The model parameters included a list of non-condiment foods consumed by at least 5 % of the girls. For each food, the parameters considered were median daily portion size (g/d), minimum and maximum frequency of intake per week, energy and nutrient content and cost per edible 100 g (GH¢/100 g). Additionally, the food group and food sub-group patterns, as well as the desired energy and population reference nutrient intake (H-AR) for the girls, were included. The 5th and 95th percentiles of the frequency of intake defined the minimum and maximum serves per week. Energy constraints and protein needs per kilogram body weight were estimated based on the median healthy BMI (22·0 kg/m^2^) and the average height of the girls utilising the FAO/WHO/UNU algorithms^(58)^. This yielded an estimated energy constraint of 2091 kcal/d for the 10–14-year age group and 2392 kcal/d for the 15–17-year age group. For fat, the average requirement of 30 % of energy was used^(59)^. The H-AR for vitamins and minerals^(50)^ were used, except for Zn, which was defined by the International Zinc Nutrition Consultative Group EAR for unrefined cereal-based diets^(52)^.

We modelled four dietary scenarios: (i) a daily diet that included foods consumed in and outside the home but without a SL from GSFP); (ii) a daily diet complemented with observed SL; (iii) a daily diet with MMB without observed SL; and (iv) daily diet complemented with observed SL and MMB. The SL programme, implemented by the GSFP, provided students with six distinct lunch recipes (online Supplementary Table S2) documented during the dietary survey period. Observed SL provided by the GSFP included (i) yoroyoro (a combination of maize and beans cooked together, accompanied by tomato sauce-stew), (ii) plain rice served with stew, (iii) rice accompanied by groundnut soup, (iv) waakye (a dish of rice and beans cooked together) served with stew, (v) jollof rice (rice prepared in a tomato sauce) and (vi) rice and beans jollof (a dish combining rice and beans cooked in a tomato sauce).

Modules I–III of Optifood were used in the analyses. Module I checked whether the model parameters can generate realistic diets for the target population and the possible range in the energy contents of diets. The two best diets – one close to the average food pattern and another that deviates from the average food pattern while remaining within the parameters of the weekly serves – were then developed for each of the four dietary scenarios using Module II. Module III was run in three phases; in Phase 1, the model minimised (worst-case) and maximised (best-case) each nutrient to estimate the robustness of the FBR in ensuring nutritionally adequate diets and determining if available foods can provide the desired nutrient levels. Problem nutrients in the optimised diets were defined as nutrients that remained below 100 % of their H-AR even when the module selected the best food sources for each of these nutrients (maximised percentage H-AR < 100 %).

In Phase 2 of Module III analysis, alternative sets of food groups, sub-groups and single nutrient-dense foods were evaluated by comparing them to the minimised (worst-case) scenario nutrient levels of the draft FBR from Phase 1. Dietary adequacy was defined as nutrient levels ≥ 100 % of the H-AR. FBR with the highest number of nutrients meeting this threshold were selected. For nutrients falling short of 100 % H-AR, those closest to this level were prioritised. We gave preference to food groups or sub-groups with the same count of nutrients meeting ≥ 100 % H-AR as individual food items, ensuring recommendations focused on broader food categories, which are easier to follow than specific food items. In Phase 3, the top FBR from Phase 2 were combined in pairs, triplicates and larger groups to create various alternative combined FBR. The combinations with the highest number of nutrients meeting the ≥ 100 % H-AR threshold were selected as the best FBR for the girls. In a sensitivity analysis, we repeated the analysis using only Modules I–II and Phase 1 of Module III analyses in Optifood to identify problem nutrients in the draft-optimised diet based on a threshold of 70 % of the WHO/FAO recommended nutrient intakes (RNI)^(60)^. These results were then compared with those obtained using the H-AR.

## Results

### Population characteristics

Out of 299 adolescent girls surveyed, we analysed the data of 292 girls, including 229 girls aged 10–14 years (early adolescence) and 63 girls aged 15–17 years (late adolescence) (Table 1). The average age of the girls was 12·0 (sd 1·4) years for early adolescents and 15·5 (sd 0·7) years for late adolescent girls. The mean weight ranged from 36·0 (sd 7·9) kg for the 10–14 years girls to 45·8 (sd 7·1) kg for the 15–17 years girls. The mean BAZ for the younger girls was slightly lower than that for the older girls; however, the older girls had a slightly lower HAZ than the younger girls. The prevalence of stunting ranged from 15·9 % for the late adolescent girls to 17·5 % for the early adolescent girls, but the prevalence of overweight/obesity was low (< 2 %) in both groups. Overall, the average Hb status was about 121 g/l, with the prevalence of anaemia slightly below 40 % for both age groups. There were about two females to one male in the households of the adolescent girls. The household literacy ratio in both groups was low, and the dependency ratio was 1·2 for girls 10–14 years old and 0·8 for girls 15–17 years old. Furthermore, on average, the household wealth index was about 48 out of a maximum score of 100, suggesting poor socio-economic status in the girls’ households. Overall, there was a high food insecurity in the households of the girls, with the prevalence of moderate-to-severe food insecurity ranging from 57·7 % among the younger age group to 60·3 % among the older girls.

**Table 1.** Population characteristics of the adolescent girls

Variable	Early adolescence (10–14 years) (*n* 229)		Late adolescence (15–17 years) (*n* 63)	
	Mean or percentage	sd	Mean or percentage	sd
Girl-level factors				
Age	12·0	1·4	15·5	0·7
Weight (kg)	36·0	7·9	45·8	7·1
Height (cm)	145·4	9·6	155·3	6·1
BMI-for-age z-score	–0·8	0·8	–0·7	0·9
Height-for-age z-score	–0·9	1·2	–1·0	0·9
Stunted (%)	17·5		15·9	
BAZ category (%)				
Thin	7·9		3·2	
Overweight/obese	1·7		1·6	
Hb status (g/l)	121.0	12.0	123.0	14.0
Anaemic (*n*, %)	36·7		38·1	
Household factors				
Household wealth index score (range: 25–100)	48·6	12·1	47·2	10·7
Household dependency ratio	1·2	0·9	0·8	0·6
Household literacy ratio	0·6	0·9	0·5	0·8
Female-to-male ratio	1·7	1·6	1·8	1·3
Household food security (%)				
Food secured	16·6		17·5	
Mild food insecurity	25·8		22·2	
Moderate food insecurity	34·1		38·1	
Severe food insecurity	23·6		22·2	

BAZ, BMI-for-age z-score.

Values are means (sd) except where specified.

### Food intake

Overall, forty-six non-condiment foods, besides six SL recipes, were modelled for each age group (online Supplementary Table S3). Foods consumed by over 90 % of the girls included whole white maize flour, onion and dried powdered pepper. The intake of cassava flour, dried okra fruit and anchovies in each age group was at least 70 %. Roasted groundnut paste, white sugar, tomato paste and refined vegetable oil were all consumed by at least 50 % of the girls. The intake rate of dark green leafy vegetables (DGLV) and fruits was low; pineapple and ebony (a wild fruit) were the only fruits consumed by one-tenth of the girls, and blackberry leaves were the only DGLV consumed by at least 5 % of the 10–14 years girls; no DGLV intake was recorded for the 15–17 years girls. Furthermore, ASF intake was also poor among the girls; anchovies and smoked fish (including mudfish, tuna and mackerel) were the only ASF consumed by the girls. The median serving sizes of all ASF were less than 10 g/d. The median serving size of food ranged from 0·3 g/d for sweet pepper to 250·8 g/d for boiled yam among the 10–14-year-old girls and 0·6 g/d for sweet pepper to 261·8 g/d for boiled yam among the 15–17-year-old girls. Most foods had serving sizes above 10 g/d among the girls (29/46 and 31/46 for the 10–14- and 15–17-year-old girls, respectively).

The most consumed SL recipes from the GSFP were rice and beans jollof and waakye (online Supplementary Table S3). Among 10–14-year-old girls, serving sizes ranged from 98 g/d for jollof rice to 170·8 g/d for rice and beans jollof. For 15–17-year-old girls, serving sizes varied from 57·4 g/d for yoroyoro (maize with beans) with stew to 285·6 g/d for rice with groundnut soup (online Supplementary Table S3).

### Energy intake and nutrient inadequacies

The study found that 10–14-year-old girls had a mean habitual energy intake of 2326·6 (sd 72·8) kcal/d, while 15–17-year-old girls had a mean habitual energy intake of 2439·1 (sd 120·7) kcal/d (Table 2). Carbohydrates accounted for approximately two-thirds (64·5 %) of the total energy intake, while proteins contributed around one-tenth (9·9 %). Fats provided just over one-fifth (21·8 %) of the energy; fibre represented roughly 4 % of the total energy intake. About three-fourths of girls had fat intake below 30 % of the energy constraint.

**Table 2.** Usual energy and nutrient intake and probability of inadequate intake among adolescent girls aged 10–17 years in Mion District, Ghana

Nutrient	Overall sample (292)			Early adolescence (*n* 229)			Late adolescence (*n* 63)		
	Mean	SE	% of inadequate intake	Mean	SE	% of inadequate intake	Mean	SE	% of inadequate intake
Energy (kcal/d)	2350·8	66·4		2326·6	72·8		2439·1	120·7	
Percentage of energy intake from the macronutrients and fibre									
Carbohydrate	64·5	5·6		64·3	5·6		64·5	5·7	
Protein	9·9	0·9		9·8	0·9		10·0	1·2	
Fat	21·8	4·8		21·9	4·6		21·5	5·6	
Fibre	3·9	0·5		3·9	0·4		3·9	0·5	
Carbohydrate (g/d)	355·5	9·2		352·1	10·8		367·8	15·2	
Protein (g/d)*	59·9	1·7	0·34	59·5	1·8	0·34	61·6	3·5	0·36
Fat (g/d)	66·0	3·14	73·8^§^	66·1	3·3	72·7^§^	65·6	6·6	77·6^§^
Fibre (g/d)	46·5	1·2		46·2	1·3		47·9	2·2	
Ca (mg/d)	390·2	14·7	99·8	381·9	16·9	99·8	420·8	22·2	99·6
Fe (mg/d)^†^	18·5	0·5	73·7	18·3	0·5	69·8	19·2	0·9	87·7
Zn (mg/d)^‡^	10·1	0·3	8·5	10·0	0·3	4·2	10·5	0·5	24·0
Vitamin A, retinol activity equivalent (µg/d)	249·5	21·3	91·5	247·4	24·0	91·7	257·2	28·6	91·1
Vitamin C (mg/d)	34·4	2·4	87·6	33·1	2·9	87·3	39·1	5·4	88·9
Thiamine (mg/d)	1·9	0·1	0·0	1·9	0·1	0·0	2·0	0·1	0·0
Riboflavin (mg/d)	0·8	0·02	96·2	0·8	0·02	95·4	0·9	0·04	99·2
Niacin (mg/d)	17·2	0·6	1·0	16·9	0·6	0·5	18·3	1·4	2·6
Vitamin B_6_ (mg/d)	2·3	0·1	2·2	2·2	0·1	2·2	2·4	0·1	2·1
Vitamin B_12_ (µg/d)	0·3	0·03	99·8	0·3	0·03	99·8	0·4	0·06	99·6
Folate, dietary folate equivalent (µg/d)	223·6	7·0	49·3	221·6	7·7	44·0	230·9	12·4	68·7
Mean probability of adequacy for micronutrients, %	44·6	2·6		45·9	2·7		39·7	3·9	

*H-AR for protein corresponds to the requirements g/kg body weight/d. ^†^The full probability approach was used for Fe. ^‡^The International Zinc Nutrition Consultative Group requirement for unrefined diets^(52)^ was used and corresponded with 9–13 years and 14–18 years girls. ^§^For fat intake, the percentage of the girls with fat intake below 30 % of the energy constraint in the Optifood model was estimated.

Almost all girls had inadequate Ca and vitamin B_12_ intake, with a similar trend observed for riboflavin and vitamins A and C. Fe inadequacy was prevalent in approximately 74 % of the girls, with an even higher rate observed in the older age group at 87·7 %. Additionally, about half of the girls had inadequate intake of folate, and the prevalence of folate inadequacy was higher for the 15–17-year-olds, where approximately two-thirds did not meet the H-AR. Also, about 8·5 % of the girls had inadequate Zn intake at the population level, but it was almost thrice among 15–17-year-old girls. Overall, the girls’ dietary intake of thiamine, niacin and vitamin B_6_ satisfied their H-AR. While protein intake was generally adequate, it was mostly from plant sources. The mean probability of adequacy for the eleven micronutrients was approximately 45 % at the population level, with a comparable pattern observed among girls aged 10–14 years. However, the MPA declined to approximately 40% among girls aged 15–17 years.

### Linear programming results

#### Draft-optimised diet

Module I generated eighteen realistic diets for the two age groups modelled in all scenarios. A draft-optimised diet (Module II) following the average dietary pattern of the 10–14-year-old girls fulfilled the requirements of six of eleven micronutrients and protein. Fat, Ca, riboflavin, vitamin B_12,_ vitamin A and Fe were problem nutrients in the optimised draft diet (Table 3). Riboflavin dietary adequacy was achieved only by adding MMB. Vitamin A requirements, marginally below 100 %, were also met through SL or MMB integration. However, Fe persistently remained only partially addressed across all diet models, reaching just over 90 % of the H-AR. Fat, Ca and vitamin B_12_ remained key problem nutrients, even though all the draft diets showed slight improvements.

**Table 3.** Draft-optimised diet (Module II) based on the average dietary pattern for four dietary scenarios for the adolescent girls in the Mion District of Ghana

Nutrient	Percentage of population reference intake* for 10–14 years girls				Percentage of population reference intake* for 15–17 years girls			
	Daily Diet^†^	Daily diet + SL	Daily diet + MMB	Daily diet + SL + MMB	Daily Diet	Daily diet + SL	Daily diet + MMB	Daily diet + SL + MMB
Protein	163·0	162·1	154·5	153·6	194·2	197·2	194·0	195·4
Fat^‡^	17·4	18·4	21·5	22·7	13·7	14·8	15·0	15·9
Ca	76·2	76·6	86·6	87·8	27·5	26·3	32·8	32·6
Fe	91·7	90·5	91·9	90·2	100·1	99·7	101·8	100·0
Zn	164·6	164·0	157·0	154·4	127·9	127·6	132·9	132·7
Vitamin A, retinol activity equivalent	97·5	100·0	100·0	100·0	89·8	91·4	100·0	100·0
Vitamin C	143·4	146·1	140·1	134·4	55·5	47·2	90·0	88·8
Thiamine	442·6	434·0	385·0	358·7	332·0	315·1	369·8	360·8
Riboflavin	85·4	84·0	117·1	113·9	64·3	61·7	100·0	100·0
Niacin	109·6	120·3	167·6	177·5	127·5	135·6	184·9	196·0
Vitamin B_6_	304·8	307·5	276·7	266·7	225·3	214·7	260·9	257·0
Folate	120·6	123·4	100·0	100·0	100·0	97·7	98·8	99·4
Vitamin B_12_	15·9	16·2	14·3	14·3	17·3	17·2	17·0	17·1
Count of nutrients ≥ 100 % of the H-AR	7	8	9	9	7	5	8	8
Cost (GH¢/d)	6·80	6·60	7·00	6·80	7·20	7·10	7·30	7·20

MMB, multiple-micronutrient-fortified biscuits; SL, school lunch from Ghana School Feeding Programme; H-AR, harmonised average requirements. *The H-AR of Allen *et al.* ^(50)^ were used except for Zn, for which the International Zinc Nutrition Consultative Group estimated average requirements^(52)^ were used. ^†^The daily diet encompasses all food consumed both at home and outside, excluding the observed school lunch provided by the Ghana School Feeding Programme. ^‡^The values for fat represent the percentage contribution to energy constraint in the models.

Similarly, for 15–17-year-old girls, adhering to their average dietary pattern also ensured protein and six out of the eleven micronutrients were addressed. Dietary inadequacies were observed in Ca, riboflavin and vitamins B_12_, A and C. Nonetheless, incorporating MMB significantly improved the intake of vitamins A and riboflavin to 100 % of the H-AR and nearly doubled vitamin C levels from 47·2 % to 90 %; however, inadequacies of Ca and vitamin B_12_ persisted, and fat also remained low in the diet.

Overall, including SL only resulted in minor increases in each nutrient constraint for both age groups. Vitamin B_12_ consistently remained a key problem nutrient for both age groups in all dietary scenarios. Analysis in the best-case scenario (Module III, Phase 1) revealed that, regardless of the dietary scenario or the addition of SL and MMB, achieving adequate vitamin B_12_ levels for both age groups was unattainable within their current dietary patterns (as illustrated in online Supplementary Tables S4 and S5). Without any modifications (Module III, Phase 1), nutrient constraints were met solely for protein in the 10–14 age group and protein and thiamine in the 15–17-year age group, as shown in online Supplementary Figures S1 and S2, respectively.

#### Nutrient-dense foods and final sets of food-based recommendations

Besides MMB, nutrient-dense foods that contributed ≥ 5 % to the H-AR in the draft-optimised diets included soybean flour, pigeon pea, sesame, groundnut paste, whole millet flour, whole white maize flour, yam tuber, blackberry leaves and dried okra fruit. Several alternative recommendations, including the nutrient-dense foods and their sub-food groups, were evaluated with combinations of SL and/or MMB to improve the worst-case scenario (online Supplementary Excel files). Among the 10–14-year-old girls, none of the best alternative sets of diets ensured dietary adequacy for vitamin B_12_ and Ca (Table 4). However, the Ca level increased by at least 4-fold for each modelled scenario compared with the worst-case scenario. Table 5 also shows that none of the best alternative sets of diets from each dietary scenario ensured dietary adequacy for Ca, vitamin B_12_ and vitamin A for 15–17-year-old girls.

**Table 4.** Evaluation of the worst-case scenario nutrient levels for the best alternative sets of food-based recommendations and diet cost for 10–14 years. Adolescent girls in Mion District, Ghana

Alternative sets of FBR	Percentage of population reference intake*													Cost/d (GH¢)	Nutrient count >= 100 % H-AR
	Protein	Fat^†^	Ca	Vit. C	Thiamine	Riboflavin	Niacin	Vit. B_6_	Folate	Vit. B_12_	Vit. A (RAE)	Fe	Zn		
No recommendation (worst-case scenario)	97·6	8·5	16·7	27·2	120·6	45·7	107·0	88·9	41·1	9·3	33·8	41·0	77·4	3·2	2
Daily diet only															
A-B-C-D-E-F-G-H-I	173·0	21·9	77·7	99·8	362·1	81·1	139·2	243·2	93·2	21·4	87·2	94·0	167·5	5·8	5
Daily food + SL															
A-B-C-D-E-F-G-H-I-J	173·0	21·9	77·7	99·8	362·1	81·1	139·2	243·2	93·2	21·4	87·2	94·0	167·5	5·8	5
Daily food + MMB															
A-B-C-D-E-F-G-H-I-K	177·0	23·2	82·2	132·9	411·0	112·0	183·4	281·0	93·2	21·4	100·8	100·3	177·1	6·0	9
Daily food + SL + MMB															
A-B-C-D-E-F-G-H-I-K-J	177·0	23·2	82·2	132·9	411·0	112·0	183·4	281·0	93·2	21·4	100·8	100·3	177·1	6·0	9

FBR, food-based dietary recommendations; RAE, retinol activity equivalent; H-AR, harmonised average requirements; SL, school lunch from Ghana School Feeding Programme, MMB, multiple-micronutrient-fortified biscuits. Vit = vitamin; A = 7 serves/week of dark green leafy vegetables; B = 7 serves/week of other vitamin A-source vegetables; C = 10 serves/week of whole maize flour; D = 10 serves of yam tuber, E = 7 serves/week of millet flour, whole; F = 14/serves/week of sesame; G = 3 serves/week of palm oil; H = 3 serves/week of fortified vegetable oil; I = 7 serves/week of smoked mudfish, J = 3 serves/week of school lunch; and K = 3 serves/week of MMB. *The H-AR of Allen *et al.* ^(50)^ were used except for Zn, for which the International Zinc Nutrition Consultative Group requirements^(52)^ were used. ^†^Represents the percentage contribution to the energy constraint.

**Table 5.** Evaluation of the worst-case scenario nutrient levels for the best alternative sets of food-based recommendations and diet cost for 15–17 years. Adolescent girls in Mion District, Ghana

Alternative sets of FBR	Percentage of population reference intake*													Cost/d	Nutrient count >= 100 % H-AR
	Protein	Fat^†^	Ca	Vit. C	Thiamine	Riboflavin	Niacin	Vit. B_6_	Folate	Vit. B_12_	Vit. A (RAE)	Fe	Zn	(GHc)	
No recommendations (worst-case scenario)	97·9	5·2	14·3	21·1	130·1	39·9	89·0	100·7	38·0	3·6	9·6	47·3	71·7	3·5	2
Daily diet															
L-B-M-N-D-G-H -O-P-Q-R	192·7	15·7	56·3	78·6	321·7	72·6	133·0	233·1	117·0	30·1	56·8	97·9	136·1	6·5	6
Daily diet + SL															
L-B-M-N-D-G-H -O-P-Q-R-J	192·7	15·7	56·3	78·6	321·7	72·6	133·0	233·1	117·0	30·1	56·8	97·9	136·1	6·5	6
Daily diet + MMB															
L-B-M-N-D-G-H -O-P-Q-R-K	198·0	18·0	65·2	131·7	397·3	121·2	205·3	302·9	117·0	30·1	83·6	108·1	151·1	6·9	9
Daily diet + MMB + SL															
L-B-M-N-D-G-H -O-P-Q-R-K-J	198·0	18·0	65·2	131·7	397·3	121·2	205·3	302·9	117·0	30·1	83·6	108·1	151·1	6·9	9

FBR, food-based dietary recommendations; SL, school lunch from Ghana School Feeding Programme; MMB, multiple-micronutrient-fortified biscuits; RAE, retinol activity equivalent; H-AR, harmonised average requirements. Vit = vitamin; L = 7 servings/week, vitamin C-rich vegetable; B = 7 servings/week, other vitamin A-source vegetable; M = 10 servings/week, pigeon pea; *N* 10 servings/week, sesame; G = 3 serving/week, palm oil; H = 3 servings/week, fortified vegetable oil; D = 10 servings/week, yam tuber; O = 14 servings/week of smoked mudfish; P = 7 servings/week, pepper dried; Q = 14 servings/week, whole maize flour; R = 3 servings/week, groundnut paste; J = 3 serving/week of school lunch; and K = 3 serving/week of MMB. *The H-AR of Allen *et al.* ^(50)^ were used except for Zn, for which the International Zinc Nutrition Consultative Group requirements^(52)^ were used. ^†^Represents the percentage contribution to the energy constraint.

Including MMB at both 3 servings/week and 5 servings/week provided comparable improvements in dietary adequacy for the girls. When incorporating MMB, there were increases in Ca by 5–10 %, Fe by 6·3–10·2 % and vitamin A by 14–27 % across the age groups of 10–14 and 15–17 years old, respectively. For both age groups, only the inclusion of MMB ensured dietary adequacy for vitamin C, riboflavin and Fe. Based on the above, the best final alternative diets for both age groups included combining their usual daily diet with SL and MMB.

Unlike MMB, including school lunch, whether at 3 or 5 servings per week, did not enhance any nutrients in the best FBR when combined with a daily diet. However, when modelled independently, SL met the dietary requirements for thiamine and niacin, with protein and vitamin B_6_ nearly reaching their H-AR at 98 and 89 %, respectively, for girls aged 10–14. A similar pattern was noted for girls aged 15–17, where SL alone satisfied the needs for thiamine and vitamin B_6_, and levels of niacin and protein approached 98 and 89 % of their respective H-AR.

In this study, the optimal alternative FBR for girls aged 10–14 met the dietary requirements for protein and eight of the eleven analysed micronutrients. However, vitamin B_12_ was still low, and folate slightly missed its target at 93·2 %. Despite significant increases, Ca levels were still below the recommended H-AR at 82·2 %. Additionally, the optimised diet did not meet the fat recommendation, achieving about 23·2 % of the desired 30 % fat constraint for energy.

**Figure 1.**
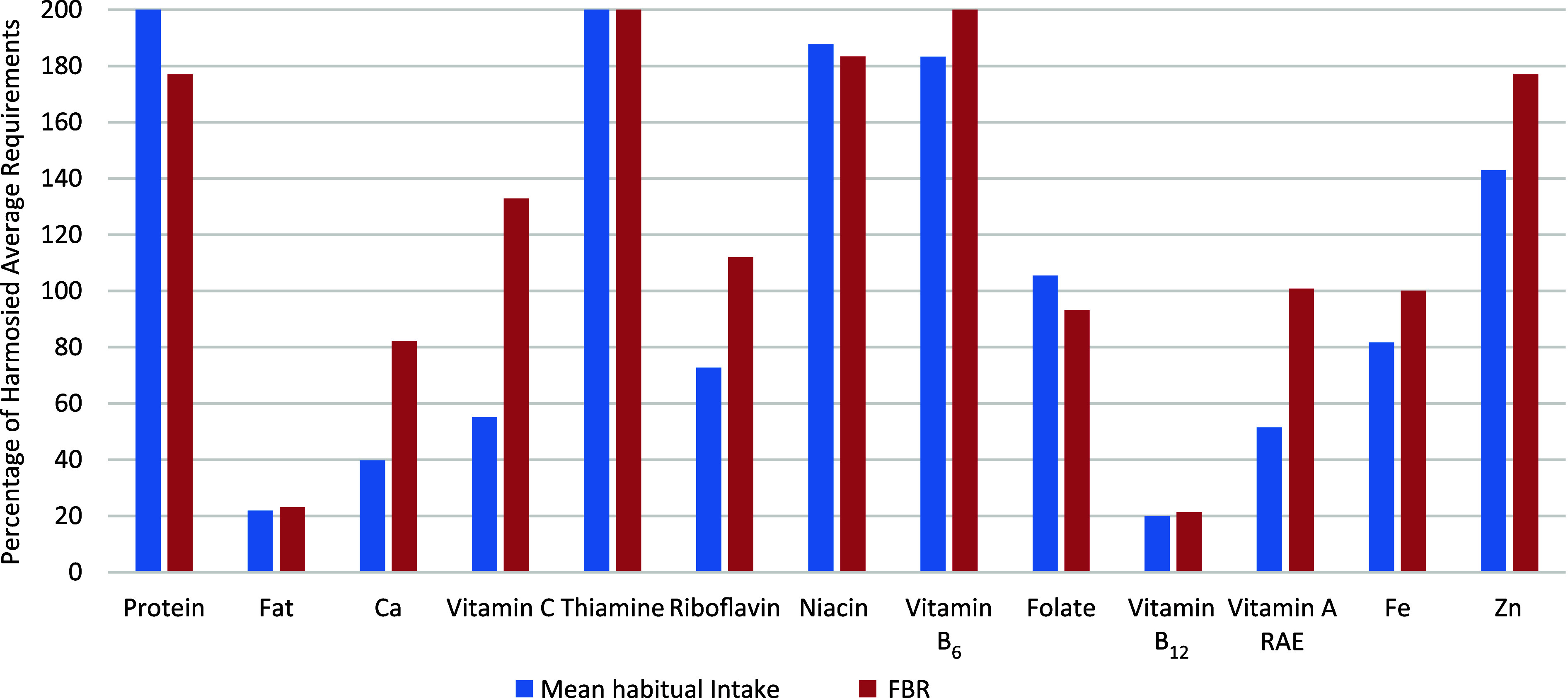
A comparison between the actual mean nutrient intake levels of the 10–14-year-old girls with the nutrient levels of the final modelled diet (worst-case scenario). Optimal levels of nutrients were capped at 200 % to improve the graph layout. For fat, the estimate represents the percentage contribution to the energy constraint. FBR, food-based dietary recommendations; RAE, retinol activity equivalent.

**Figure 2.**
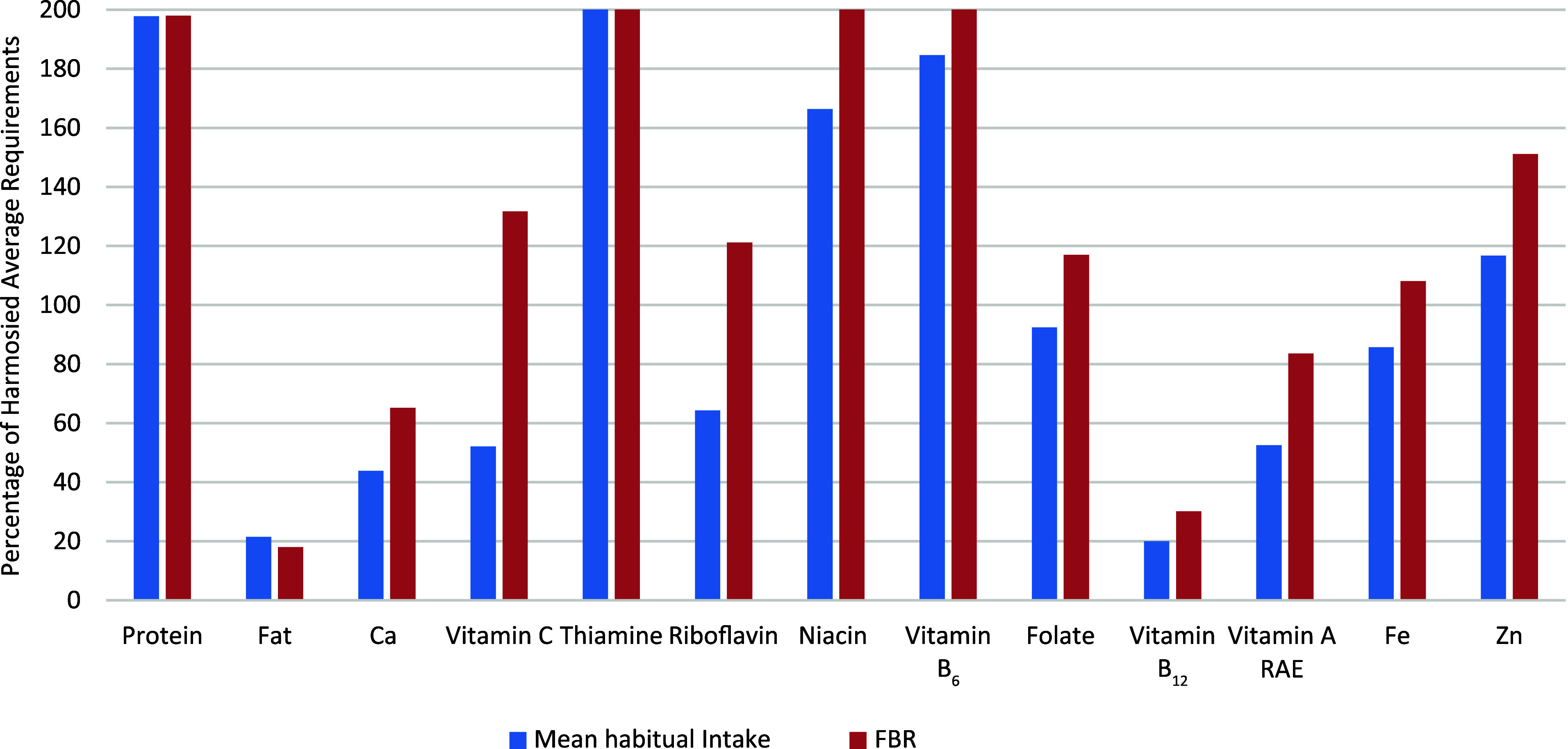
A comparison between the actual mean nutrient intake levels of the 15–17-year-old girls with the nutrient levels of the final modelled diet (worst-case scenario). Optimal levels of nutrients were capped at 200 % to improve the graph layout. For fat, the estimate represents the percentage contribution to the energy constraint. FBR, food-based dietary recommendations; RAE, retinol activity equivalent.

The scenario for girls aged 15–17 years was similar: the optimal alternative FBR satisfied the nutrient requirements for protein and eight of the eleven modelled micronutrients. This age group also saw low vitamin B_12_ and Ca levels, achieving only 30·1 and 65·2 % of their respective H-AR. Despite the addition of MMB, which raised vitamin A levels by approximately 27 %, it remained below the H-AR at 83·6 %. The fat content was also inadequate, reaching only 18 % of the desired 30 % fat constraint for energy. However, Figs. 1 and 2 illustrate that the selected final FBR improved micronutrient intake for both age groups, although vitamin B_12_ levels remained comparable to observed habitual intake.

Table 6 outlines the weekly servings from different food groups or sub-groups in the optimal FBR across dietary scenarios. For girls aged 10–14, the best alternative FBR included 3 servings per week each of palm oil and fortified vegetable oil; 7 weekly servings each of DGLV, other vitamin A-rich vegetables and boneless fish; 17 servings/week of cereals and grains; 10 servings/week of starchy roots and tubers; 14 servings/week of nuts and seeds; and 3 weekly servings each of SL and MMB. For girls aged 15–17, the best alternative diet also consisted of 3 weekly servings each of palm oil and fortified vegetable oil; 7 servings/week each of other vitamin A-rich vegetables, vitamin C-rich vegetables and other vegetables; 14 weekly servings of cereals and grains and boneless fish; 10 servings/week each of legumes and starchy roots and tubers; 13 servings/week of nuts and seeds; and 3 servings/week each of SL and MMB.

**Table 6.** Best sets of alternative food-based recommendations (Module III, Phase 3) and diet cost for 10–17-year-old adolescent girls in Mion District, Ghana

Food group^1^	Number of weekly serves for 10–14 years girls				Number of weekly serves for 15–17 years girls			
	Daily Diet	Daily diet + SL	Daily diet + MMB	Daily diet + SL + MMB	Daily Diet	Daily diet + SL	Daily diet + MMB	Daily diet + SL + MMB
Added fat	6	6	6	6	6	6	6	6
Red palm oil	3	3	3	3	3	3	3	3
Fortified vegetable oil	3	3	3	3	3	3	3	3
Staples	27	27	27	27	24	24	24	24
Cereals and grains	17	17	17	17	14	14	14	14
Starchy roots tubers	10	10	10	10	10	10	10	10
Legumes, nuts and seeds	14	14	14	14	23	23	23	23
Nuts and seed	14	14	14	14	13	13	13	13
Legumes					10	10	10	10
Vegetables	14	14	14	14	21	21	21	21
DGLV	7	7	7	7				
Vitamin A source, other vegetables	7	7	7	7	7	7	7	7
Vitamin C-rich vegetables	–	–	–	–	7	7	7	7
Other vegetables	–	–	–	–	7	7	7	7
Animal-sourced food	7	7	7	7	14	14	14	14
Fish without bone	7	7	7	7	14	14	14	14
School lunch	N/A	3	N/A	3	N/A	3	N/A	3
MMB	N/A	N/A	3	3	N/A	N/A	3	3
Count of nutrients ≥ 100 % of the H-AR	5	5	9	9	6	6	9	9
Cost (GH¢/d)	5·8	5·8	6·0	6·0	6·5	6·5	6·9	6·9

SL, school lunch from Ghana School Feeding Programme; MMB, multiple-micronutrient-fortified biscuits; DGLV, dark green leafy vegetables; H-AR, harmonised average requirements. Food groups or sub-groups without any recommendations are not shown; N/A, not applicable.

#### Sensitivity analyses

In a sensitivity analysis using WHO/FAO RNI, Fe and folate emerged as key problem nutrients in the draft-optimised diet for both age groups, with Zn levels also falling below adequacy, suggesting that these nutrients were sensitive to the constraints applied (online Supplementary Table S6).

## Discussion

In the present study, we assessed nutrient inadequacies and evaluated whether and to what extent the inclusion of a fortified food product such as MMB to local FBR with(out) regular SL from the GSFP improves dietary adequacy. Although energy and protein intake were adequate, we observed a high prevalence of inadequate intake of several micronutrients, including Ca, vitamins B_12_, riboflavin, vitamins A, C, Fe, folate and Zn in the diet of girls in the Mion District of Ghana. The observed nutrient inadequacies confirm the need for nutrition interventions to improve the dietary adequacy of adolescent girls in rural settings. Similar results have been reported for adolescent girls in Senegal^(15)^ and in the Ashanti Region of Ghana^(16)^. The prevalence of inadequate intake was higher for the 15–17-year-olds. The multiple nutrient inadequacies are unsurprising, as this is usually observed in predominantly cereal-consuming populations^(61)^.

The final FBR for the girls were a combination of their usual daily diet complemented with MMB and SL. For both age groups, the best alternative FBR ensured dietary adequacy for eight of the eleven micronutrients modelled. Vitamin B_12_ and Ca remained key problem nutrients for both age groups, with folate marginally low and vitamin A considerably low for the 10–14- and 15–17-year-old girls, respectively. Nevertheless, the optimal FBR notably enhanced the girls’ dietary adequacy compared with the observed habitual diet, although vitamin B_12_ and fat intake remained close to their observed habitual intake.

In all dietary scenarios in the FBR, the modelled energy intake from fat was below the constraint for the girls, particularly for those aged 15–17. In contrast, the observed habitual energy intake from fat (22 %) was within the recommended range of 20–30 % by the FAO^(59)^. Given the recent trend of overweight and obesity, it is reasonable that the FBR do not prioritise meeting fat constraints. Unfortunately, there is a lack of comparative studies describing the diet of rural Ghanaian adolescents. However, similar findings were reported in one study among school-aged children^(17)^ and another among infants and young children^(31)^ in the region.

ASF are valuable sources of vitamin A, Ca and vitamin B_12_^(62)^. However, in this study, incorporating ASF or their sub-groups within the typical portion size constraints of the adolescent girls did not substantially enhance the dietary adequacy of the modelled diets. Although fish was commonly consumed, the smaller portion sizes (< 10 g/d), particularly for fish consumed with bones, limited its influence on the nutrient constraints. Dairy products are known to enhance Ca intake^(63)^; however, less than 3 % of participants reported consuming canned evaporated milk – the sole dairy product noted in the 24HR, with a maximum weekly intake frequency of approximately once. Consequently, including evaporated milk in the models did not enhance the adequacy of the optimised diets. Nevertheless, including mudfish improved Ca and folate by at least 5 % for both age groups, with a higher weekly serving being more beneficial for the older age group. While Ca was considered a constraint in the recently launched Ghana FBDG, vitamin B_12_ was not identified as a problem in the population^(64)^, which apparently may not be true for some vulnerable groups in Ghana, such as adolescents. Therefore, national food intake data, segregated by various sub-groups, are needed to assess nutrient inadequacies across the population. These data will help revise the Ghana FBDG to address specific nutrient inadequacies in targeted populations.

Fe and Zn are frequently identified as critical problem nutrients in contexts similar to this study^(31,65)^. While Fe and Zn inadequacies were prevalent among the girls, achieving dietary adequacy with the existing dietary pattern was feasible, a finding that was consistent with Talsma *et al.* ^(32)^ among school-aged children in Eastern Kenya. Several food items contributed substantially to Fe and Zn, including cowpeas, pigeon peas, sesame, groundnuts, soybeans and yams. Although these foods are rich in phytates, we factored this into the nutrient constraints for Fe and Zn. The substantial contributions of these foods may be attributed to their large portion sizes and high weekly consumption frequencies within the population. However, it must be stated that Fe, folate and Zn were sensitive to the nutrient constraints used; they emerged as problem nutrients when the WHO/FAO RNI were applied in sensitivity analyses. While the EAR is the standard for assessing population-level nutrient adequacy, the absence of EAR for many nutrients in FAO/WHO references^(60)^ has led to alternatives, such as 70 % of the RNI. In conformity with Allen *et al.* ^(50)^, who argue that RNI are unsuitable for population-level assessments, we adopted the H-AR. The H-AR are conceptually aligned with the EAR. Using 100 % H-AR as the cutoff, we modelled diets that meet nutrient requirements similar to the EAR, consistent with approaches in recent studies^(66–69)^.

Our results indicate that dietary adequacy for Ca and vitamin B_12_ cannot be achieved within the confines of the current dietary patterns, and drastic modifications in food intake patterns are necessary, but this may not be practical. The best-case scenario nutrient levels (Module III, Phase 1) indicated the potential for achieving adequate Ca levels. However, the consistently low Ca levels in both observed and modelled diets could be attributed to insufficient ASF intake. Purposefully selecting foods to satisfy the Ca requirement also compromised the levels of other nutrients that are of public health concern. Alternative strategies, including biofortified foods, fortified foods, nutrient supplements and, more recently, edible insects, appear critical to achieving complete dietary adequacy^(32,65,70)^.

In our study, the girls’ intake of DGLV and other vitamin A-rich vegetables was low. Blackberry leaves were the only DGLV included in the model, but only for the 10–14-year-old age group. Our study was conducted in December–January, coinciding with the early part of the dry season. The dry season is generally characterised by a lower intake of DGLV and vitamin A-rich vegetables in the region^(71)^. Furthermore, the intake of vitamin C-rich fruits and vegetables was poor among the girls. It is important to highlight that the dry season presents the most challenging period. Nevertheless, achieving nutrient or dietary adequacy during this time suggests that dietary needs can also be met in the rainy season when fruits and vegetables are abundant. Therefore, our findings underscore the importance of implementing additional strategies to ensure dietary adequacy year-round. Developing and promoting food preservation techniques, such as drying, could help make seasonal fruits and vegetables available year-round, thereby addressing nutrient gaps for nutrients such as vitamins A and C. However, this would require investment in infrastructure, training and awareness to make these strategies more accessible and feasible for local communities.

In the present study, we found that the inclusion of 3–5 servings/week of MMB without any other recommendations achieved adequacy for protein, thiamine, niacin and vitamin B_6_, indicating still some nutrient gaps. However, when combining MMB (3 or 5 servings/week) with the alternative set of FBR, dietary adequacy was additionally reached for riboflavin, vitamin C and Fe. However, this represented only a marginal increase, besides a noticeable improvement in Ca and vitamin A, particularly for girls in late adolescence. With a 5–10 % increase in Ca and no change in vitamin B_12_, both remain critical problem nutrients in the girls’ diet. This finding indicates a possible mismatch between the nutrient content of the MMB and the needs of this population. In our population, biscuits were not commonly consumed, and when not provided for free during interventions, they are relatively costly, making adherence to such a recommendation in real-life settings unrealistic. Biscuits are also not a healthy dietary option, particularly given the emerging challenges of overweight and obesity among adolescents. Consequently, the GSFP programme should consider incorporating fortified foods to substantially improve dietary adequacy beyond its current focus.

Contrary to expectations and existing literature^(17,24)^, including observed SL from the GSFP in the model did not improve the dietary adequacy of the FBR. However, when analysed independently, SL met the dietary requirements for thiamine and niacin in younger girls and thiamine and vitamin B_6_ in older girls. It also contributed significantly to the H-AR for protein and either vitamin B_6_ or niacin in the respective age groups. This may be due to the similarity between SL recipes from the GSFP and the girls’ usual diets. Overall, SL from the GSFP appears inadequate in improving dietary adequacy for critical micronutrients lacking in adolescent girls’ usual diets. A recent study on the GSFP in the Greater Accra Region found that meals typically included only two to three out of five recommended food groups, primarily starchy staples, pulses/nuts/seeds and occasionally vegetables, with minimal inclusion of ASF and no reported fruit in meals^(72)^. This highlights the need for substantial menu revisions to incorporate nutrient-dense foods, particularly ASF, such as milk, eggs, meat and fish. The GSFP seems more focused on benefits like school attendance, retention and attention in class^(73,74)^ rather than dietary improvements. A more cost-effective approach might involve offering the standard SL three times a week, supplemented with specialised fortified foods on the other 2 d.

The modelled FBR, tailored to our target population’s specific dietary patterns and needs, largely align with the Ghana FBDG^(27)^, except for the fruit and ASF intake, which was not applicable in our study due to low intake. Notably, the recommendations concerning staples, legumes and nuts, vegetables and fats were consistent with the national dietary guidelines. It is crucial to recognise that while these FBR deviate slightly from the dietary patterns observed in the study population, the Ghana FBDG serve as broad guidelines for what the population should consume to remain healthy and do not specifically cater to the patterns noted in our study population. Overall, this research addresses nutrient inadequacies in a specific vulnerable group by evaluating the effect of integrating fortified snacks, such as MMB and regular SL from the GSFP, to fill observed nutrient gaps not met by their regular diets. In contrast, the Ghana FBDG offer dietary guidelines for the general population, including adolescents, but do not specifically address the role of fortified foods in preventing nutrient inadequacies.

To our knowledge, this is the first study to extensively study the diet of rural Ghanaian adolescent girls and to formulate FBR with Optifood for this specific population group. Despite rigorous training and standard procedure for the 24HR, recall bias is still a problem for the 24HR. It should be noted that the developed FBR are not only population-specific but also context-specific for rural adolescent girls in northern Ghana.

### Conclusion

In conclusion, despite widespread micronutrient inadequacies identified in the diets of girls aged 10–17 in Ghana’s Mion District, optimised FBR through the Optifood linear programming tool, demonstrated potential in achieving dietary adequacy for protein and most micronutrients, except Ca and vitamin B_12_, with substantial improvements in vitamin A and folate, which also remained below the H-AR. MMB emerged as a critical intervention, improving the intake of riboflavin, vitamins C and A and a marginal increase in Fe, highlighting the essential role of fortified foods in bridging nutrient gaps. There is a need to revise the prevailing GSFP lunch menus to include nutrient-dense foods, particularly targeting Ca and vitamin B_12_ gaps; incorporating ASF like milk, eggs, fish, meat and fortified foods would improve dietary adequacy. Our study highlights that a cost-effective and comprehensive strategy to improve dietary adequacy for adolescent girls in rural areas in Northern Ghana could involve integrating a regular SL offered three times a week with specialised fortified foods on the remaining days.

## Supporting information

Azupogo et al. supplementary material 1 Azupogo et al. supplementary material

Azupogo et al. supplementary material 2 Azupogo et al. supplementary material

Azupogo et al. supplementary material 3 Azupogo et al. supplementary material

## Supplementary material

For supplementary material/s referred to in this article, please visit https://doi.org/10.1017/S0007114525103929.
