# Supplementary material for: Nutrient gaps and dietary adequacy among adolescent girls in rural North-Eastern Ghana: the role of local food-based approaches, school lunch and multiple-micronutrient fortified biscuits
Source: Br J Nutr. 2025 Jul 10;134(2):134–46. doi: 10.1017/S0007114525103929 (PMC12433747; doi:10.1017/S0007114525103929)
Supplement: Azupogo et al. supplementary material 1 — Azupogo et al. supplementary material [file S0007114525103929sup001.pdf]

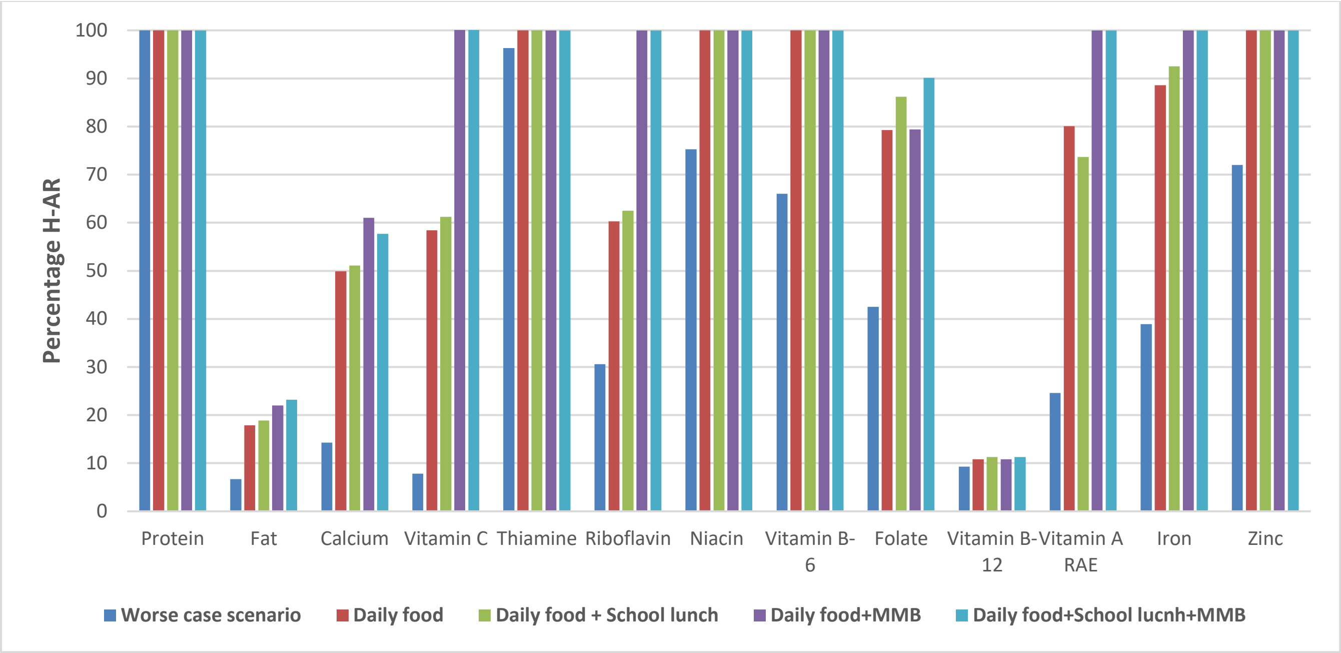

Figure S1: Comparison of the percentage population reference intake (H-AR) coverage for the best diet in four dietary scenarios (FBRs based on: (1) locally available food- "daily foods"; (2) daily food + school lunch; (3) daily food +MMB and (4) daily food +school lunch+MMB) against the worst-case scenario nutrient levels among 10-14 yrs. adolescent girls in the Mion District, Ghana. *Values above the dashed line 100% indicate dietary adequacy. Values above 100 were capped at 100. Values for fat represent the percentage contribution to the energy constraint.*
