# Supplementary material for: Nutrient gaps and dietary adequacy among adolescent girls in rural North-Eastern Ghana: the role of local food-based approaches, school lunch and multiple-micronutrient fortified biscuits
Source: Br J Nutr. 2025 Jul 10;134(2):134–46. doi: 10.1017/S0007114525103929 (PMC12433747; doi:10.1017/S0007114525103929)
Supplement: Azupogo et al. supplementary material 3 — Azupogo et al. supplementary material [file S0007114525103929sup003.docx]

**Table S1: Nutrient Composition of Multiple-Micronutrient Fortified Biscuits (MMB) in the Ten2Twenty-Ghana RCT**

| **No.** | **Nutrient** | **Product name** | **Nutrient content of fortified biscuits (mg) per serving (51.3g)** |
| --- | --- | --- | --- |
| 1 | Vitamin A | Dry vitamin A palmitate | 0.504 |
| 2 | Vitamin D | Dry vitamin D3 | 0.005 |
| 3 | Vitamin E | Dry vitamin E | 6.00 |
| 4 | Vitamin K | Dry vitamin K1 | 0.05 |
| 5 | Thiamine | Thiamine mononitrate | 1.20 |
| 6 | Riboflavin | Riboflavin | 1.20 |
| 7 | Niacin | Niacinamide | 14.00 |
| 8 | Vitamin B6 | Pyridoxine Hydrochloride | 1.60 |
| 9 | Folic acid | Folic acid | 0.311 |
| 10 | Vitamin B12 | Vitamin B12 | 0.002 |
| 11 | Ascorbic acid | Ascorbic acid | 70.00 |
| 12 | Calcium | Calcium carbonate | 150 |
| 13 | Cupper | Cupper Gluconate | 0.20 |
| 14 | Iodine | Potassium Iodide | 0.04 |
| 15 | Iron | Ferrous Fumarate | 4.05 |
| 16 | Magnesium | Magnesium oxide | 52.50 |
| 17 | Selenium | Sodium Selenite | 0.012 |
| 18 | Zinc | Zinc Oxide | 2.38 |

**Table S2: School Lunch (SL) Recipes from the Ghana School Feeding Programme, December 2018–January 2019, Mion District, Ghana**

| **Recipe Code** | **Food Code** | **Dish name** | **Components (if any)** | **Ingredients** | **The mean weight of the ingredient** | **Proportion of ingredient** |
| --- | --- | --- | --- | --- | --- | --- |
| 348 |  | **Rice and beans Jollof** | **No side dishes** | **Overall dish Total** | **78094.5** |  |
| 348 | 18 | Rice and beans Jollof |  | Local Rice | 32794.7 | 0.42 |
| 348 | 156 | Rice and beans Jollof |  | Beans, white, black eye | 8467.7 | 0.11 |
| 348 | 63 | Rice and beans Jollof |  | Anchovies | 208.7 | 0.003 |
| 348 | 88 | Rice and beans Jollof |  | Dawadawa | 126.0 | 0.002 |
| 348 | 45 | Rice and beans Jollof |  | Tomatoes, fresh | 1633.3 | 0.02 |
| 348 | 85 | Rice and beans Jollof |  | Vegetable oil | 3766.7 | 0.05 |
| 348 | 86 | Rice and beans Jollof |  | Bouillon cube | 83.3 | 0.001 |
| 348 | 216 | Rice and beans Jollof |  | Pepper, fresh | 54.7 | 0.001 |
| 348 | 317 | Rice and beans Jollof |  | Salt, non-iodized | 612.5 | 0.01 |
| 348 | 321 | Rice and beans Jollof |  | Saltpetre (potassium nitrate) | 42.9 | 0.001 |
| 348 | 39 | Rice and beans Jollof |  | Onions | 439.1 | 0.01 |
| 348 | 307 | Rice and beans Jollof |  | Curry powder | 80.0 | 0.001 |
| 349 |  | **Yoroyoro** |  | **Overall dish Total** | **98384.9** |  |
| 349 |  | Yoroyoro | **Maize and beans** | **Total weight** | **87080.8** |  |
| 349 | 318 | Yoroyoro |  | maize | 23926.4 | 0.24 |
| 349 | 156 | Yoroyoro |  | Beans, white, black eye | 11724.3 | 0.12 |
| 349 | 317 | Yoroyoro |  | Salt | 226.3 | 0.002 |
| 349 | 321 | Yoroyoro |  | Saltpetre (potassium nitrate) | 576.0 | 0.01 |
| 349 |  | Yoroyoro | **Tomato sauce/stew** | **Total weight** | **11304.1** |  |
| 349 | 84 | Yoroyoro |  | Frytol, cooking oil | 2803.5 | 0.03 |
| 349 | 82 | Yoroyoro |  | Palm oil | 1930.4 | 0.020 |
| 349 | 39 | Yoroyoro |  | Onions | 287.4 | 0.003 |
| 349 | 63 | Yoroyoro |  | Anchovies | 123.5 | 0.001 |
| 349 | 86 | Yoroyoro |  | Maggi cube | 96.0 | 0.001 |
| 349 | 317 | Yoroyoro |  | Salt, non-iodized | 70.0 | 0.001 |
| 349 | 216 | Yoroyoro |  | Pepper | 70.1 | 0.001 |
| 349 | 45 | Yoroyoro |  | Tomatoes, fresh | 1466.7 | 0.02 |
| 349 | 321 | Yoroyoro |  | Saltpetre (potassium nitrate) | 325.8 | 0.003 |
| 349 | 56 | Yoroyoro |  | Kulikulizim (dried groundnut powder)/KKZ | 86.4 | 0.001 |
| 353 |  | **Waakye** |  | **Overall dish Total** | **64393.7** |  |
| 353 |  | Waakye | **Rice and beans** | **Total weight** | **51932.8** |  |
| 353 | 18 | Waakye |  | Local rice | 15778.8 | 0.25 |
| 353 | 156 | Waakye |  | Black eye beans | 6190.0 | 0.10 |
| 353 | 321 | Waakye |  | Saltpetre (potassium nitrate) | 43.5 | 0.001 |
| 353 | 316 | Waakye |  | Salt iodized | 185.6 | 0.003 |
| 353 |  | Waakye | **Tomato sauce/stew** | **Total weight** | **12461.0** |  |
| 353 | 45 | Waakye |  | Tomatoes paste | 2225.3 | 0.04 |
| 353 | 63 | Waakye |  | Anchovies | 347.6 | 0.005 |
| 353 | 216 | Waakye |  | Pepper, fresh chilli | 50.3 | 0.001 |
| 353 | 85 | Waakye |  | Vegetable oil | 2461.7 | 0.04 |
| 353 | 39 | Waakye |  | Onions, fresh | 205.3 | 0.003 |
| 353 | 86 | Waakye |  | Bouillon cube | 120.0 | 0.002 |
| 353 | 316 | Waakye |  | Salt iodized | 93.0 | 0.001 |
| 354 |  | **Jollof** | **No side dishes** | **Overall dish Total** | **79322.0** |  |
| 354 | 18 | Jollof |  | Local rice | 28281.6 | 0.36 |
| 354 | 45 | Jollof |  | Tomatoes paste | 2144.0 | 0.03 |
| 354 | 63 | Jollof |  | Anchovies | 151.7 | 0.002 |
| 354 | 86 | Jollof |  | Bouillon cube | 140.0 | 0.002 |
| 354 | 85 | Jollof |  | Vegetable oil | 2790.4 | 0.04 |
| 354 | 39 | Jollof |  | Onion, fresh | 244.1 | 0.003 |
| 354 | 86 | Jollof |  | Bouillon cube | 86.0 | 0.001 |
| 354 | 216 | Jollof |  | Pepper, fresh | 6973.4 | 0.09 |
| 354 | 316 | Jollof |  | Salt iodized | 6325.0 | 0.08 |
| 355 |  | **Plain rice** |  | **Overall dish Total** | **58820.7** |  |
| 355 |  | Plain rice | **Plain rice** | **Total weight** | **53726.8** |  |
| 355 | 18 | Plain rice |  | Local rice | 14140.8 | 0.24 |
| 355 | 317 | Plain rice |  | Salt, non-iodized | 129.5 | 0.002 |
| 355 |  | Plain rice | **Tomato sauce/stew** | **Total weight** | **5093.9** |  |
| 355 | 84 | Plain rice |  | Vegetable oil | 2315.2 | 0.04 |
| 355 | 45 | Plain rice |  | Tomato paste | 1500.0 | 0.03 |
| 355 | 135 | Plain rice |  | Fish herrings smoked | 147.1 | 0.003 |
| 355 | 317 | Plain rice |  | Salt, non-iodized | 83.0 | 0.001 |
| 355 | 216 | Plain rice |  | Pepper, dried powder | 64.8 | 0.001 |
| 355 | 86 | Plain rice |  | Bouillon cube | 25.0 | 0.0001 |
| 355 | 39 | Plain rice |  | Onion, fresh | 303.0 | 0.01 |
| 356 |  | **Rice with Groundnut soup** |  | **Overall dish Total** | **69438.7** |  |
| 356 |  | Rice with Groundnut soup | **Plain rice** | **Total weight** | **43295.7** |  |
| 356 | 18 | Rice with Groundnut soup |  | Local rice | 17727.7 | 0.26 |
| 356 | 317 | Rice with Groundnut soup |  | Salt, non-iodized | 259.7 | 0.004 |
| 356 |  | Rice with Groundnut soup | **Groundnut soup** | **Total weight** | **26143.0** |  |
| 356 | 58 | Rice with Groundnut soup |  | Groundnut paste | 5919.6 | 0.09 |
| 356 | 45 | Rice with Groundnut soup |  | Tomato paste | 1600.0 | 0.02 |
| 356 | 317 | Rice with Groundnut soup |  | Salt, non-iodized | 148.0 | 0.002 |
| 356 | 63 | Rice with Groundnut soup |  | Anchovies | 72.0 | 0.001 |
| 356 | 86 | Rice with Groundnut soup |  | Bouillon cube | 68.0 | 0.001 |
| 356 | 216 | Rice with Groundnut soup |  | Pepper, dried powder | 57.4 | 0.001 |
| 356 | 39 | Rice with Groundnut soup |  | Onion, fresh | 237.1 | 0.003 |
| 356 | 122 | Rice with Groundnut soup |  | Gabo (fermented onion leaves) | 22.3 | 0.0003 |

**Table S3: Food groups and foods used for modelling as consumed by the study population of adolescent girls aged 10-17 years, Mion District Ghana, Dec 2018-Jan 2019**

| **Food group and food item** | **Early adolescent girls (229)** | | | | **Late adolescent girls (n=63)** | | | | **Cost (GH¢)/100g** |
| --- | --- | --- | --- | --- | --- | --- | --- | --- | --- |
|  | **% consuming food item** | **Median daily servings (g/day)** | **Min serves per week^1^** | **Max serves per week** | **% consuming food item** | **Median daily servings (g/day)** | **Min serves per week** | **Max serves per week** |  |
| **Added fat** |  |  | **2** | **21** |  |  | **0** | **21** |  |
| Vegetable oil, refined | 50.8 | 19.8 | 0.5 | 7 | 46.2 | 18.0 | 0 | 7 | 1.03 |
| Vegetable oil, frytol | 39.7 | 8.3 | 1 | 7 | 40.2 | 9.1 | 0 | 7 | 1.03 |
| Groundnut oil | 16.7 | 12.5 | 0 | 7 | 26.5 | 20.8 | 0 | 7 | 1.00 |
| Shea butter | 7.1 | 19.5 | 0 | 7 | N/A | N/A | N/A | N/A | 1.01 |
| Palm oil, red | 6.4 | 11.8 | 1 | 7 | 12.1 | 5.3 | 0 | 7 | 1.23 |
| Margarine (fortified) | N/A | N/A | N/A | N/A | 5.3 | 2.1 | 0 | 7 | 2.5 |
| **Added sugar** |  |  | **1** | **7** |  |  | **0** | **7** |  |
| Sugar white/brown | 54.8 | 20.0 | 0 | 7 | 52.3 | 25.8 | 0 | 7 | 0.62 |
| **Grains & grain products** |  |  | **7** | **35** |  |  | **7** | **35** |  |
| Maize flour, whole grain white, RTboiled | 92.1 | 125.8 | 3 | 28 | 93.8 | 131.9 | 3 | 28 | 1.23 |
| Maize dough, whole grain white RTboiled | 18.3 | 61.2 | 1 | 28 | 16.7 | 63.2 | 1 | 28 | 0.22 |
| Rice, local brown unpolished raw, RTboiled | 42.9 | 111.2 | 2 | 28 | 36.4 | 109.5 | 2 | 28 | 0.47 |
| Guinea corn flour, whole grain red, RTboiled | 34.9 | 36.7 | 0.5 | 28 | 21.2 | 31.2 | 0.5 | 28 | 0.37 |
| Guinea corn dough, whole grain red, RTboiled | 8.7 | 39.7 | 0.5 | 28 | 9.1 | 42.9 | 0.5 | 28 | 0.27 |
| Millet flour whole grain, RTboiled | 5.6 | 19.6 | 0 | 28 | N/A | N/A | N/A | N/A | 0.83 |
| Spaghetti, macaroni dried, RTboiled | 15.1 | 8.3 | 0 | 7 | 25.0 | 12.3 | 0 | 7 | 0.90 |
| **Starchy roots & other starchy plant foods** |  |  | **3** | **21** |  |  | **0** | **21** |  |
| Cassava flour, RTboiled | 77.0 | 56.1 | 0 | 7 | 74.2 | 49.1 | 0 | 21 | 1.39 |
| Yam tuber raw, RTboiled | 33.3 | 250.8 | 0 | 7 | 29.6 | 261.8 | 1 | 21 | 0.37 |
| Yam tuber flour, RTsteamed | 11.9 | 51.5 | 0 | 7 | 19.7 | 51.5 | 0 | 21 | 0.37 |
| Yam tuber raw, RTroasted | 7.1 | 229.5 | 0 | 7 | 7.6 | 226.1 | 0 | 21 | 0.51 |
| **Legumes, nuts & seeds** |  |  | **3.5** | **28** |  |  | **0** | **28** |  |
| Beans white dried raw, RTboiled | 34.9 | 38.3 | 1 | 14 | 28.0 | 46.7 | 1 | 14 | 0.62 |
| Sesame seeds roasted, RTboiled | 18.3 | 22.4 | 0.5 | 14 | 15.2 | 16.6 | 0 | 14 | 1.43 |
| Beans soya flour whole, RTboiled | 17.5 | 18.9 | 0 | 7 | 13.6 | 30.4 | 0 | 14 | 0.26 |
| Groundnut whole, RTroasted | 16.7 | 25.9 | 0 | 21 | 6.1 | 25.9 | 0 | 14 | 1.12 |
| Groundnut roasted paste, RTboiled, fried | 63.5 | 20.5 | 0.5 | 21 | 64.4 | 23.1 | 0 | 14 | 0.85 |
| Melon seeds raw, RTboiled | 6.4 | 10.3 | 0.5 | 21 | 7.6 | 10.3 | 0 | 14 | 0.70 |
| Pigeon peas dried, RTboiled | N/A | N/A | N/A | N/A | 7.6 | 44.8 | 0 | 14 | 0.18 |
| Koose, RTfried | 6.4 | 63.9 | 0.5 | 14 | 7.6 | 45.7 | 0 | 14 | 0.82 |
| **Meat, fish & eggs** |  |  | **7** | **21** |  |  | **3** | **21** |  |
| Fish anchovies smoked, RTboiled | 83.3 | 5.8 | 3 | 7 | 77.3 | 5.2 | 0 | 14 | 3.59 |
| Fish mackerel raw, RTsmoked, boiled | 15.1 | 2.2 | 1 | 14 | 15.2 | 2.2 | 0 | 14 | 1.19 |
| Fish tuna raw, RTsmoked | 11.9 | 1.6 | 1 | 14 | 18.9 | 1.6 | 0 | 14 | 1.51 |
| Fish mud smoked, RTboiled | 14.3 | 7.5 | 2 | 14 | 25.8 | 9.3 | 0 | 14 | 0.63 |
| **Fruits** |  |  | **0** | **14** |  |  | **0** | **14** |  |
| Ebony fruit | 12.7 | 31.0 | 0 | 7 | 15.9 | 49.6 | 0 | 7 | 0.30 |
| Pineapple raw | 10.3 | 3.6 | 0 | 7 | 10.6 | 2.8 | 0 | 7 | 0.56 |
| **Vegetables** |  |  | **14** | **56** |  |  | **14** | **63** |  |
| Bra fruit, white dried, RTboiled | 18.3 | 5.2 | 1 | 42 | 16.7 | 4.2 | 2 | 42 | 0.43 |
| Blackberry leaves, raw, RTboiled | 5.6 | 23.1 | 1 | 7 | N/A | N/A | N/A | N/A | 0.26 |
| Okra fruit, dried powder, RTboiled | 83.3 | 9.2 | 2 | 42 | 89.4 | 10.5 | 3 | 42 | 1.43 |
| Tomato paste concentrate, RTboiled | 54.8 | 15.8 | 1 | 7 | 53.8 | 14.0 | 0 | 7 | 1.57 |
| Tomato raw, RTboiled | 17.5 | 22.6 | 2 | 7 | 18.9 | 25.0 | 1 | 7 | 0.48 |
| Pepper, red dried, RTboiled | 95.2 | 2.4 | 1 | 42 | 95.7 | 2.7 | 0 | 42 | 3.00 |
| Pepper chilli, raw, RTboiled | 18.3 | 3.1 | 1 | 42 | 24.2 | 3.1 | 1 | 42 | 3.02 |
| Pepper sweet green raw, RTboiled, stewed | 18.3 | 0.3 | 1 | 42 | 12.9 | 0.4 | 0 | 42 | 0.64 |
| Onion bulb, raw, RTboiled | 88.9 | 8.2 | 1 | 42 | 90.2 | 8.5 | 1 | 42 | 0.86 |
| Onion bulb, raw, RTdeepfried | 21.4 | 3.0 | 1 | 42 | 18.9 | 5.0 | 0 | 42 | 0.86 |
| Ginger fresh, RTboiled | 57.9 | 2.4 | 1 | 42 | 50.8 | 2.6 | 1 | 42 | 1.03 |
| Ginger fresh, RTdeepfried | 7.1 | 0.9 | 1 | 42 | 7.6 | 0.6 | 0 | 42 | 1.03 |
| **Bakery & breakfast cereals** |  |  | **0** | **7** |  |  | **0** | **7** |  |
| Bread, sugar | 5.6 | 98.0 | 0 | 7 | 13.6 | 97.9 | 0 | 7 | 0.77 |
| Bread, tea | 7.9 | 102.0 | 0 | 7 | 9.1 | 102.0 | 0 | 7 | 0.74 |
| Biscuit, sweet | 7.9 | 31.0 | 0 | 7 | 7.1 | 24.8 | 0 | 7 | 1.64 |
| **Sweetened snacks & desserts** |  |  | **0** | **7** |  |  | **0** | **7** |  |
| Candy | 11.1 | 7.5 | 0 | 7 | 12.1 | 10.0 | 0 | 7 | 2.91 |
| Ice cream | 5.6 | 70.0 | 0 | 7 | 7.6 | 66.7 | 0 | 7 | 9.05 |
| **Savoury Snacks** |  |  | **0** | **7** |  |  | **0** | **7** |  |
| Wheat flour white raw, RTfried | 7.1 | 46.9 | 0 | 7 | 6.1 | 38.2 | 0 | 7 | 0.72 |
| **School Lunch^2^** |  |  | **3** | **7** |  |  | **3** | **7** |  |
| Rice and beans jollof recipe | 7.1 | 168.0 | 0.5 | 7 | 2.3 | 170.8 | 0.5 | 7 | 0.38 |
| Jollof dish recipe | 1.59 | 145.5 | 0.5 | 7 | 0.8 | 98.0 | 0.5 | 7 | 0.41 |
| Waakye with stew recipe | 1.59 | 177.8 | 0.5 | 7 | 9.1 | 152.6 | 0.5 | 7 | 0.38 |
| Yoroyoro with stew recipe^3^ | N/A | N/A | 0.5 | 7 | 0.8 | 57.4 | 0.5 | 7 | 0.24 |
| Rice plain with stew recipe ^3^ | N/A | N/A | 0.5 | 7 | 0.8 | 113.4 | 0.5 | 7 | 0.41 |
| Rice with Groundnut Soup recipe^3^ | N/A | N/A | 0.5 | 7 | 0.8 | 285.6 | 0.5 | 7 | 0.29 |

N/A, not applicable; ^1^All instances of zero (0) at the group level were adjusted to 0.001 since zero returns an error in Optifood.  ^2^School feeding recipes from the Ghana School Feeding Programme were included even when they did not meet the 5% criteria; ^3^The School feeding recipes were included for the age group 11-14 years, although none consumed the recipes. RT, retention factor applied.

**Table S4: *Best-case scenario percentage nutrient level for the diet of 10-14-year-old girls in the Mion district, Ghana***

|  | **Daily food** | **Daily food + School lunch** | **Daily food + MMB** | **Daily food + School lunch + MMB** |
| --- | --- | --- | --- | --- |
| Protein | 297.4 | 297.7 | 295.6 | 297 |
| Fat | 156.6 | 163.6 | 164.9 | 172.7 |
| Calcium | 130.6 | 130.3 | 139.8 | 142.5 |
| Vitamin C | 191.1 | 204.3 | 274 | 287.6 |
| Thiamine | 483.9 | 476.2 | 577.1 | 569.2 |
| Riboflavin | 118.2 | 116 | 189.2 | 189 |
| Niacin | 361.7 | 364.4 | 445.8 | 448.5 |
| Vitamin B-6 | 337.4 | 335.9 | 414.5 | 411.7 |
| Folate | 184.7 | 183.6 | 182.8 | 181.1 |
| Vitamin B-12 | 52.2 | 52.6 | 52.5 | 53.8 |
| Vitamin A RAE | 149.8 | 156.8 | 183.7 | 191.9 |
| Iron | 126.3 | 125.1 | 133.1 | 131.9 |
| Zinc | 213.6 | 212.5 | 224.4 | 223.4 |

**Table S5: Best-case scenario percentage nutrient level for the diet of 15-17-year-old girls in the Mion district, Ghana**

|  | **Daily food** | **Daily food + School lunch** | **Daily food + MMB** | **Daily food + School lunch + MMB** |
| --- | --- | --- | --- | --- |
| Protein | 291.4 | 292.1 | 290 | 290.7 |
| Fat | 131.8 | 137.6 | 139.1 | 144.7 |
| Calcium | 99.1 | 99.7 | 109.2 | 109.7 |
| Vitamin C | 159.1 | 166.2 | 225.4 | 232.5 |
| Thiamine | 432.1 | 427 | 504.7 | 499.5 |
| Riboflavin | 106.6 | 105.1 | 162.4 | 160.3 |
| Niacin | 314.6 | 332.5 | 395.2 | 408.5 |
| Vitamin B-6 | 342.6 | 340.3 | 412.6 | 410.3 |
| Folate | 188.6 | 188.4 | 187.3 | 186.8 |
| Vitamin B-12 | 47.2 | 47.5 | 47.2 | 47.4 |
| Vitamin A RAE | 155.3 | 161.9 | 188.7 | 195.3 |
| Iron | 132.7 | 131.6 | 138.4 | 136.0 |
| Zinc | 178.6 | 178.3 | 187.5 | 187.0 |

**Table S6: Draft Optimized Diet (Module II) Based on the Average Dietary Pattern of Adolescent Girls in the Mion District, Ghana, Using WHO/FAO Recommended Nutrient Intake as the Population Reference Standard**

| **Nutrient** | **10-14 years Girls** | **15-17 years Girls** |
| --- | --- | --- |
|  | **Daily diet + SL + MMB with WHO/FAO RNIs as nutrient reference** | **Daily diet + SL + MMB with WHO/FAO RNIs as nutrient reference** |
| Protein | 136.9 | 158.5 |
| Fat^3^ | 20.1 | 18.8 |
| Calcium | 60.9 | 31.7 |
| Iron | 76.1 | 38.3 |
| Zinc | 83.2 | 86.6 |
| Vitamin A RAE | 96.8 | 95.9 |
| Vitamin C | 272.0 | 145.7 |
| Thiamine | 299.7 | 293.4 |
| Riboflavin | 139.4 | 134.5 |
| Niacin | 102.8 | 128.6 |
| Vitamin B-6 | 341.2 | 290.6 |
| Folate | 60.3 | 46.8 |
| Vitamin B-12 | 10.2 | 14.2 |
| Count of nutrients ≥70% RNIs | 9 | 8 |
| Cost (GH¢/day) | 7.7 | 7.3 |

SL, school-lunch from the Ghana School-Feeding Programme; MMB-Multiple-micronutrient fortified biscuits , RNI, recommended nutrient intake
